# Protective Role of Mitochondrial Uncoupling Proteins against Age-Related Oxidative Stress in Type 2 Diabetes Mellitus

**DOI:** 10.3390/antiox11081473

**Published:** 2022-07-28

**Authors:** Maša Čater, Lidija Križančić Bombek

**Affiliations:** Institute of Physiology, Faculty of Medicine, University of Maribor, Taborska ulica 8, 2000 Maribor, Slovenia

**Keywords:** uncoupling proteins, reactive oxygen species, aging, age-related diseases, diabetes

## Abstract

The accumulation of oxidative damage to DNA and other biomolecules plays an important role in the etiology of aging and age-related diseases such as type 2 diabetes mellitus (T2D), atherosclerosis, and neurodegenerative disorders. Mitochondrial DNA (mtDNA) is especially sensitive to oxidative stress. Mitochondrial dysfunction resulting from the accumulation of mtDNA damage impairs normal cellular function and leads to a bioenergetic crisis that accelerates aging and associated diseases. Age-related mitochondrial dysfunction decreases ATP production, which directly affects insulin secretion by pancreatic beta cells and triggers the gradual development of the chronic metabolic dysfunction that characterizes T2D. At the same time, decreased glucose oxidation in skeletal muscle due to mitochondrial damage leads to prolonged postprandial blood glucose rise, which further worsens glucose homeostasis. ROS are not only highly reactive by-products of mitochondrial respiration capable of oxidizing DNA, proteins, and lipids but can also function as signaling and effector molecules in cell membranes mediating signal transduction and inflammation. Mitochondrial uncoupling proteins (UCPs) located in the inner mitochondrial membrane of various tissues can be activated by ROS to protect cells from mitochondrial damage. Mitochondrial UCPs facilitate the reflux of protons from the mitochondrial intermembrane space into the matrix, thereby dissipating the proton gradient required for oxidative phosphorylation. There are five known isoforms (UCP1-UCP5) of mitochondrial UCPs. UCP1 can indirectly reduce ROS formation by increasing glutathione levels, thermogenesis, and energy expenditure. In contrast, UCP2 and UCP3 regulate fatty acid metabolism and insulin secretion by beta cells and modulate insulin sensitivity. Understanding the functions of UCPs may play a critical role in developing pharmacological strategies to combat T2D. This review summarizes the current knowledge on the protective role of various UCP homologs against age-related oxidative stress in T2D.

## 1. Introduction

Mitochondria are the organelles of the cell that are responsible for energy production. Mitochondria are essential for aerobic ATP synthesis by oxidative phosphorylation and for the synthesis of heme, cholesterol, and phospholipids, as well as for apoptosis and cell signaling [1]. They are unique cell organelles because they have their own genome. Mitochondrial DNA (mtDNA) can self-replicate and transcribe. Because mtDNA is small and circular, it only encodes proteins essential for normal oxidative phosphorylation, namely, some subunits of the mitochondrial respiratory chain and some tRNA and rRNAs for the assembly of the mitochondrial translational machinery. The nuclear genome encodes all other proteins necessary for proper mitochondrial function, which are then imported into the mitochondria [2].

Mitochondria are also the largest source of reactive oxygen species (ROS) in the cell, which are generated when electrons leak during respiration [3]. At levels that are non-damaging, ROS are involved in important signal transduction pathways related to cell growth, apoptosis, kinase activation, immune responses, gene expression regulation, and calcium signaling [4,5,6,7,8,9,10]. However, excessive amounts of ROS not only directly damage lipids, proteins, and DNA, resulting in mtDNA mutations [11] but also affect a variety of stress-sensitive intracellular signaling pathways, such as the mitogen-activated protein kinase (MAPK) pathway, Jun amino-terminal kinase/stress-activated protein kinase (JNK/SAPK) pathway, and the nuclear factor kappa B (NF-kB) pathway [12,13,14,15,16]. Increased expression of the gene products of these pathways causes additional cellular damage [17,18]. mtDNA damage can impair viability and various cellular functions, and maintaining its integrity with age is crucial for survival [19]. Accordingly, mitochondrial dysfunction has been associated with various age-related diseases, such as type 2 diabetes (T2D), neurodegenerative diseases, cancer, and cardiovascular diseases [20,21,22,23,24].

T2D is a disease characterized by insufficient production of insulin, excessive secretion of glucagon by pancreatic beta cells, and insulin resistance, resulting in impaired energy metabolism in the pancreas, liver, skeletal muscle, and other organs [25]. Data for 2021 show that the global prevalence of T2D in 20- to 79-year-olds is 10.5%. The prevalence is lowest in young adults aged 20–24 years (2.2%) and steadily increases to 24% in elderly individuals aged 75–79 years. Projections for 2045 are similar, except that the percentages will be slightly higher in each age group. Most importantly, the aging of the world population will result in a higher proportion of people with T2D over the age of 60 [26,27], along with a higher incidence of cardiovascular complications and metabolic syndrome. The increased incidence of various comorbidities and the simultaneous use of different medications, which may lead to drug interactions in older diabetic patients, make the management of T2D particularly complex and challenging. Therefore, new approaches for controlling T2D are needed, including individualized treatment strategies [28].

Although the primary cause of T2D has not yet been determined, mitochondrial dysfunction in the organs responsible for insulin secretion (pancreatic beta cells), in the target organs of insulin action (skeletal and cardiac muscle cells and liver cells), and in the target organs associated with the major complications of T2D (kidneys, retina, nerves, and vascular cells) may play an important role in the pathophysiology of the disease [29]. Since ATP is critical for the production and release of insulin, altered mitochondrial bioenergetics associated with impaired glucose and fatty acid metabolism have been linked to defects in insulin and glucagon secretion in T2D [30].

UCPs are a group of five homologous proteins located in the inner mitochondrial membrane of various tissues. They are involved in several tasks and cellular functions, from thermoregulation to modulation of insulin secretion and neuroprotection [31,32,33,34]. The most diverse spectrum of UCPs is found in the mitochondria of skeletal muscle, which express all five UCPs. For this reason, skeletal muscle is one of the best-studied tissues in regard to advancing our knowledge of UCP function and associated pathologies [35,36]. UCPs have been intensively studied in the last three decades because of their involvement in glucose and lipid metabolism [37,38,39,40,41,42,43]. In addition, many studies in mice, rats, and humans have shown that mitochondrial uncoupling proteins (UCPs) have important protective effects against oxidative stress and mitochondrial dysfunction [44,45,46]. However, their exact role has not been fully elucidated.

In this review, we highlight some significant associations between different UCP homologs and T2D and emphasize the importance of UCPs as potential pharmacological targets in the treatment of T2D.

## 2. Mitochondria, ROS, and Oxidative Stress

**Figure 1:**
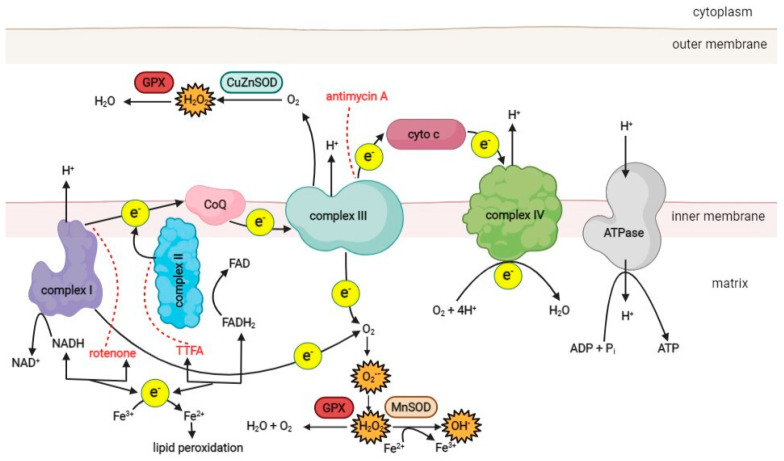
A schematic overview of the ROS production pathways in the respiratory chain of the mitochondrion. (CoQ–coenzyme ubiquinol, cyto c–cytochrome c, MnSOD–manganese superoxide dismutase, CuZnSOD–copper-zinc superoxide dismutase, GPX–glutathione peroxidase, TTFA–thenoyltrifluoroacetone). Red dotted lines represent the inhibition effect of specific compounds on the respiratory chain complexes. Created with BioRender.com.

The main source of ROS in the cell is the mitochondrial respiratory chain, which consists of four protein complexes responsible for generating the proton motive force across the inner mitochondrial membrane (Figure 1). Complex I (NADH-ubiquinone oxidoreductase) accepts electrons from NADH and passes them to complex II (succinate dehydrogenase), which oxidizes succinate to fumarate. As an enzyme of the Krebs cycle, complex II provides a direct link between the Krebs cycle and the respiratory chain [47]. Electrons from complexes I and II are transferred to ubiquinone (Q), which is then oxidized by complex III (ubiquinol cytochrome C oxidoreductase). Finally, electrons are passed to complex IV (cytochrome C oxidase) and used to reduce molecular O_2_ as the final electron acceptor, producing water. As electrons are transferred through the respiratory chain to complexes I, III, and IV, protons from NADH and FADH_2_ are translocated from the mitochondrial matrix into the intermembrane space, generating a strong proton motive force that subsequently drives the mitochondrial ATPase to produce ATP [3,47].

Under physiological conditions, 1–5% of the oxygen consumed by the mitochondria is incompletely reduced to superoxide (O_2_^•−^), the primary ROS species formed in mitochondria, mainly in complexes I and III [48,49]. Other sources of ROS in the cell include NAD(P)H oxidase, various isoforms of nitric oxide synthase (NOS), xanthine oxidase, and lipoxygenases [6,50].

Superoxide is a charged molecule and, as such, does not readily diffuse across membranes. However, mitochondrial ROS can enter the cytosol after conversion to hydrogen peroxide (H_2_O_2_) by superoxide dismutase (SOD) [19]. There are three known SOD isoforms. SOD 1 (copper-zinc SOD; CuZn-SOD) is located in the mitochondrial intermembrane space, cytosol, and nucleus. SOD 2 (manganese SOD; Mn-SOD) is found only in the mitochondrial matrix, while SOD 3 (extracellular CuZn-SOD; EC-SOD) is present in the extracellular space [51]. In the mitochondrial matrix, H_2_O_2_ is reduced to water by catalase and glutathione peroxidase [52,53]. However, in the presence of transition metals such as copper or iron, H_2_O_2_ can be converted to reactive and damaging hydroxyl radicals (^•^OH) via the Fenton reaction or the Haber–Weiss reaction [6]. The resulting ROS can damage the proteins, lipids, and DNA of the cell. ROS generation, oxidative damage, and antioxidant defense mechanisms of the cell have been discussed in detail elsewhere [11,14,54].

Mitochondrial dysfunction, such as that associated with electron transport blockade, causes the respiratory chain to enter a highly reduced state. This triggers increased electron leakage and the production of superoxide anions and other ROS that further damage the cell’s biomolecules in a destructive cycle that can lead to progressive cell function degeneration and, eventually, cell death.

Mitochondria are not only the main producers of ROS but also their main target. In differentiated, nondividing cells, mtDNA is constantly replicating as intracellular ROS generation progresses. Oxidative stress in the form of various oxygen radicals modifies DNA. The damage leads to single- and double-strand breaks and base changes, resulting in cellular dysfunction, mutagenesis, and even carcinogenesis [19]. In particular, hydroxyl radicals are known to attack guanine bases [55]. One of the most common DNA lesions caused by ROS-induced mutagenesis is the modified guanine base 8-oxoguanine, which pairs equally efficiently with adenine and cytosine [19] and causes transversion mutations.

Since mitochondrial ROS production is much higher than that in the cytoplasm, ROS-induced damage to mtDNA is much more significant than damage to nuclear DNA. The mutation rate of mtDNA is up to twenty times higher than that of nuclear DNA, and its point mutation rate is more than two orders of magnitude higher than that of nuclear genes [56,57]. In addition, mitochondria tend to accumulate toxic xenobiotics. The matrix side of the mitochondrial membrane has a negative potential. It attracts lipophilic cations, including drugs and biotoxic chemicals, and causes their massive concentration, leading to exogenously induced mitochondrial damage [58,59,60]. Mutations in mtDNA accumulate with age and can lead to cellular dysfunction [19,61,62]. Large mtDNA deletions have been detected in healthy elderly humans and other species, such as *Caenorhabditis elegans*, mice, rats, and monkeys [63,64,65,66,67]. Moreover, an increased frequency of mitochondrial genomic deletions in brain samples has been associated with Huntington’s disease and Alzheimer’s disease [68,69].

Cells use various antioxidant systems to degrade ROS. One of the most important antioxidant enzymes in mitochondria is glutathione peroxidase [6,70]. Its function is to remove hydrogen peroxide, which is formed from superoxide anions (Figure 1). In addition, vitamin E, present in the inner mitochondrial membrane, acts as an antioxidant by accepting unpaired electrons and generating a stable product [71]. The oxidative damage repair system in mitochondria plays an important role in normal cellular function. It includes enzymes that repair oxidized mtDNA, eliminate mutant dNTPs, and degrade damaged mtDNA [72,73,74]. In humans, the *MTH1* gene encodes 8-oxo-dGTPase, a human counterpart of the well-studied *Escherichia coli* protein MutT, which is essential for the removal of adenine paired with 8-oxoguanine in DNA [75]. Studies on the accuracy of mitochondrial DNA polymerase gamma in mtDNA replication and proofreading have shown that it is comparable to nuclear DNA polymerase [76], suggesting that higher mtDNA mutation rates result from more severe damage or/and weaker post-replication repair activities. Since dNTPs for mtDNA synthesis are synthesized inside mitochondria, all oxidized dNTPs must be removed in situ.

In addition to naturally occurring enzymatic and non-enzymatic antioxidants, mitochondria have endogenously regulated proteins called uncoupling proteins (UCPs) that can limit oxidative damage to cells.

## 3. Mitochondrial Dysfunction in T2D

Blood glucose levels must be adequately regulated to meet the energy needs of tissues while preventing excessive blood glucose levels from damaging blood vessel walls and nervous system cells. Blood glucose levels are controlled by two types of pancreatic islet cells: beta cells, which secrete insulin and amylin, and alpha cells, which secrete glucagon [77]. Insulin primarily causes cells to take up glucose from the blood and store it as glycogen or fat. Insulin also inhibits the mobilization of glucose from glycogen, protein, and fat stores [78]. Amylin released by beta cells inhibits alpha cells from producing glucagon [79]. Amylin and insulin are released by beta cells when blood glucose levels are high and inhibit the production of glucagon. Conversely, a fall in blood glucose levels causes the production of insulin and amylin by beta cells to be reduced, allowing alpha cells to produce glucagon unimpeded. The hormone glucagon increases blood glucose levels by causing the liver to break down glycogen stores and stimulating the formation of glucose from other small molecules through gluconeogenesis [79].

T2D is characterized by impaired pancreatic beta cell function and insulin resistance [80]. To maintain normal plasma glucose levels, the pancreas secretes more insulin in the early stages of the disease due to insulin insensitivity of peripheral tissues. As the disease progresses and pancreatic function deteriorates, insulin can no longer maintain glucose at a homeostatic level. As a result of the decreased responsiveness of the liver to insulin and abnormalities in the regulation of glucagon secretion, hepatic glucose production increases [81]. Along with decreased glycogen uptake and impaired insulin secretion, these events lead to hyperglycemia. As tissues become resistant to insulin, the pancreas compensates by producing more insulin, resulting in hyperinsulinemia. Another metabolic dysfunction that accompanies T2D is dyslipidemia, a condition characterized by abnormal lipid levels in the blood and a major risk factor for cardiovascular disease in T2D patients. Several processes are involved in T2D-associated dyslipidemia, including hyperglycemia, impaired lipid metabolism, and increased triglyceride synthesis as a result of insulin resistance [82]. Together, hyperglycemia, hyperinsulinemia, and dyslipidemia are important contributors to the increased oxidative stress associated with T2D and related pathologies [83].

Superoxide production rate depends on the concentration of potential electron donors and the local O_2_ concentration. In isolated mitochondria, significant O_2_^•−^ production was observed under two conditions. First, when ATP production was low with consequently high proton motive force and a reduced coenzyme Q (CoQ) content; second, when the NADH/NAD^+^ ratio in the mitochondrial matrix was high [3]. The latter is particularly prominent in intense lipid or glucose metabolism, resulting in subsequent ROS generation and chronic diabetic complications [84].

Increased oxidative stress plays an important role in the onset and progression of T2D, as evidenced by increased levels of oxidative stress markers and reduced antioxidant levels in diabetic subjects [85]. Several mechanisms contribute to oxidative stress under diabetic conditions. These include disruption of the mitochondrial electron transport chain [86], increased activity of the polyol pathway [87], glucose autooxidation [88], and formation of advanced glycation end products (AGEs) [89]. Interestingly, in T2D patients and their first-degree relatives, serum levels of copper and iron, two potent prooxidant trace elements, have been found to be elevated and correlate with increased glycated hemoglobin levels [90]. Copper has the potential to increase the formation of ROS during the conversion of Cu(I) to Cu(II) [91]. In addition, under hyperglycemic conditions, iron and copper participate in glucose autooxidation that yields hydrogen peroxide, which undergoes further metal-catalyzed conversion to form the highly reactive hydroxyl radical [92]. Increased production of ROS and ROS-related cellular damage can also result from high-dose pharmaceutical iron supplementation, such as in anemic pregnant women, leading to gestational diabetes [93]. To combat excess ROS generation, cells produce antioxidants, as evidenced by their increased levels in blood and saliva samples from diabetic patients [94].

By promoting insulin resistance, impaired glucose tolerance, and mitochondrial dysfunction, oxidative stress further contributes to the progression of diabetes and associated pathologies. The contribution of oxidative stress and mitochondrial dysfunction to T2D is further examined in the following sections.

### 3.1. ROS-induced Metabolic and Biochemical Changes in T2D

During the development of T2D, metabolic and biochemical changes gradually accumulate. In parallel with various polygenic causes, a cascade of successive events leads to the accumulation of defects in the mitochondrial oxidative phosphorylation machinery and mitochondrial fatty acid beta-oxidation. The resulting accumulation of triglycerides in muscle and liver cells leads to insulin resistance [95,96,97]. In addition, diabetes-associated ROS and oxidative stress stimulate various signaling cascades. The polyol pathway is induced, AGE formation progresses, the hexosamine pathway is upregulated, and protein kinase C isoform activation increases [12,98], which impairs insulin signaling and leads to insulin resistance [12].

Only small amounts of glucose are metabolized through the polyol pathway under normal conditions. However, under hyperglycemic conditions, hexokinase is saturated, leading to an increase in glucose concentration and its entry into the polyol pathway [99]. In the case of diabetes, the polyol pathway is increased in tissues where insulin is not essential for glucose uptake into cells, such as the kidneys, retina, and peripheral nerves [87]. These changes in the polyol pathway lead to a reductive imbalance as the intracellular NAD(P)H concentration decreases and the NADH concentration increases, which then serves as a substrate for NADH oxidase to produce more ROS. The reduction in NAD(P)H significantly impairs the antioxidant system by decreasing the level of the antioxidant glutathione in cells because its activity is highly dependent on NAD(P)H [100]. NAD(P)H reduction also impairs the synthesis of nitric oxide, which is known as a vasoprotective agent and an excellent quencher of superoxide anions [101,102].

In addition, intracellular and extracellular AGEs are formed under hyperglycemic conditions. The production of excess ROS is induced via AGE receptor binding, which activates protein kinase C isoforms, the NF-kB pathway, and NADPH oxidase [103]. This leads to alterations in MAPK cascades [12], which include important signaling pathways regulating cell proliferation, differentiation, apoptosis, and stress responses. ROS-activated NF-kB in pancreatic beta cells eventually leads to beta-cell apoptosis [104].

Normally, only a small amount of fructose-6-phosphate is channeled away from the glycolytic pathway of glucose metabolism. However, in diabetes, the intracellular glucose concentration is increased, and a larger amount of fructose-6-phosphate leaves glycolysis. Under hyperglycemic conditions, elevated mitochondrial superoxide production inhibits GAPDH activity, leading to an enhancement of the hexosamine pathway and an accumulation of glycolytic intermediates [105]. Moreover, the enhanced hexosamine pathway is an additional source of ROS. Accordingly, in patients with T2D who are insulin-resistant, the levels of the rate-limiting enzyme of the hexosamine pathway glutamine-fructose-6-phosphate aminotransferase (GFAT) were found to be elevated. This suggests a role for increased activity of the hexosamine pathway in glucose toxicity and insulin resistance [106]. Together, increased formation and expression of AGE receptors, activation of the polyol pathway and protein kinase C isoforms, and upregulation of the hexosamine pathway lead to the progression and exacerbation of T2D.

### 3.2. Response of Pancreatic Beta Cells to Hyperglycemia and Elevated ROS Production

Normal insulin secretion from pancreatic beta cells follows a biphasic pattern driven by underlying oscillatory changes in intracellular calcium concentration [107,108,109]. The first calcium and insulin peak is followed by a brief decrease, which is then superseded by a sustained plateau phase with superimposed fast calcium oscillations [110]. Insulin secreted in the first phase rapidly lowers postprandial blood glucose levels as it first passes through the liver. In contrast, second-phase insulin travels to more distant organs and remains elevated as long as the stimulus persists [111]. With aging and T2D, biphasic kinetics and the total amount of insulin secreted are impaired [112,113,114]. In mouse islets exposed to a glucotoxic medium, insulin secretion was reduced in the first phase, whereas secretion started much earlier in the second phase [115]. This pattern differs markedly from the normal biphasic calcium activity of beta cells in healthy tissues [116], where even a supraphysiological glucose concentration elicits a marked biphasic response [117]. Changes in biphasic activity have also been confirmed in human islet cells under glucotoxic conditions [115].

Altered insulin secretion as a result of T2D and in old age has been attributed to impaired mitochondrial metabolism leading to a decrease in ATP production in beta cells [30]. The result is altered function of ATP-dependent potassium channels, decreased depolarization of beta cells, and decreased glucose-dependent insulin secretion [118]. Since mitochondrial function is critical for coupling insulin secretion to glucose metabolism in beta cells by controlling the ATP:ADP ratio, decreased mitochondrial ATP generation also contributes to insulin resistance [96].

Hyperglycemia promotes oxidative stress in several ways, including increasing the activity of enzymes involved in the production of ROS, such as xanthine oxidase [119], and the accumulation of AGEs, which impair the activity of antioxidant enzymes [120]. Chronic hyperglycemia increases glucose metabolism, which depletes NAD^+^ with enzymes such as glyceraldehyde-3-phosphate dehydrogenase, aldose reductase, and sorbitol dehydrogenase, reducing their availability to the antioxidant enzymes SOD 2 and reduced glutathione (GSH), which also require NAD^+^ [121,122]. The combined effects of increased ROS generation in mitochondrial oxidative metabolism and decreased antioxidant capacity lead to the accumulation of ROS, which is exacerbated by ceramide synthesis due to excessive insulin signaling [123].

Along with the decreased activity of antioxidant enzymes, increased levels of DNA damage markers and protein and lipid peroxidation products can be observed in hyperglycemic conditions [12]. As in other organs, the increased extracellular glucose concentration resulting from diminished glucose uptake into cells negatively affects insulin-secreting beta cells in pancreatic islets of Langerhans, which have a reduced ability to adapt to glucotoxicity. Indeed, pancreatic beta cells are more sensitive to ROS and reactive nitrogen species (RNS) because their antioxidant levels are lower than in other tissues [124,125,126].

In addition, ROS have been shown to induce stress signaling pathways in beta cells, including the NF-kB signaling pathway and the JNK signaling pathway [18]. This increased stress signaling has been associated with the suppression of insulin production, possibly by decreasing the DNA-binding activity of pancreatic duodenal homeobox 1 (PDX-1), which is critical for proper pancreatic beta cell function [127,128]. In addition, hexosamine pathway activation in beta cells suppresses PDX-1 binding to genes involved in insulin secretion [129]. Together, these processes contribute to beta cell dysfunction and subsequent impairment of insulin production in T2D.

### 3.3. T2D-Related Mitochondrial Dysfunction Contributes to Various Diabetes-Related Pathologies

In chronic hyperglycemia, the overproduction of ROS suppresses cellular enzymatic and non-enzymatic antioxidant mechanisms in various tissues, increasing oxidative stress [12,130]. Increased ROS production causes substantial damage, especially in tissues with rich vasculature and high energetic demands [131]. Indeed, excessive ROS production triggered by hyperglycemia and ensuing hyperinsulinemia is a common denominator of many T2D-associated complications, including those affecting the vascular system, retina, kidneys, and brain [21].

The risk for T2D and various comorbidities, including microangiopathies and macroangiopathies, increases with age, sedentary lifestyle, and unhealthy diet. Oxidative stress resulting from excessive ROS production and decreased antioxidant capacity affects vascular endothelial function, extracellular matrix formation, and smooth muscle cell growth and migration [132]. It is, therefore, not surprising that high ROS levels lead to dysfunction of organs with sensitive capillary networks, which is the main cause of diabetic retinopathy [133] and diabetic nephropathy [134]. Excess superoxide generation associated with T2D can directly inhibit endothelial enzymes such as endothelial nitrogen oxide synthase (eNOS) [135], which has potent antiatherogenic effects, protecting against diabetic vasculopathy. In diabetic eNOS knockout mice, hypertension with arteriolar hyalinosis and microaneurysms developed early in life, leading to high mortality. These pathologies were accompanied by albuminuria and renal insufficiency due to increased glomerular and peritubular capillaries. These histological changes could be improved by insulin therapy [136,137].

Moreover, hyperglycemia increases ROS production in vascular endothelial cells [138]. It induces damage via at least three independent biochemical pathways, namely, glucose-induced activation of protein kinase C (PKC pathway) [139], increased formation of advanced glycation end products (AGEs pathway) [140], and enhanced glucose metabolism via the aldose reductase pathway (polyol pathway) [12,141].

There is evidence that inhibition of eNOS activity or signaling leading to diabetic macrovascular dysfunction may underlie chronic coronary heart disease in T2D patients [142]. In diabetic cardiomyopathy, adverse structural and functional tissue remodeling is associated with enhancement or impairment of multiple biochemical pathways caused by hyperglycemia-induced ROS overproduction [143]. The role of ROS in the pathogenesis of diabetic cardiomyopathy is well documented [144,145]. For instance, transgenic overexpression of catalase or manganese superoxide dismutase in diabetic mice was associated with partial restoration of mitochondrial function and cardiomyocyte contractility [146,147]. Other studies have also demonstrated increased myocardial production of NADPH oxidase-derived ROS in diabetic rats [100,148]. These results suggest that oxidative stress associated with diabetic cardiomyopathy originates from mitochondrial and extramitochondrial sources.

Diabetic retinopathy is a complication of diabetes that impacts retinal blood vessels. It is thought to be caused by changes in the structure and function of retinal blood vessels in response to chronic hyperglycemia and the ensuing increased formation of AGEs and increased oxidative stress [133]. AGEs have been shown to promote the formation of new blood vessels (neovascularization) in the retina and increase the permeability of existing blood vessels, both of which are hallmarks of diabetic retinopathy [149].

ROS are also important sources of damage to the retina and, if not removed by antioxidant systems, can cause irreparable damage [150], leading to blindness. The contribution of oxidative stress to diabetic retinopathy is supported by the observation that levels of oxidative stress markers are increased in the retina of patients with diabetes [151] and in diabetic animals [152]. Accordingly, at least in animal models, antioxidant supplementation was reported to improve retinal structure and function [153,154,155].

In the brain, high ROS levels resulting from hyperglycemia are strongly associated with the development of diabetic neuropathy by causing direct damage to the nerves or by affecting the blood vessels that supply the nerves [143,156]. The accumulation of ROS and the decreased ability of neurons to eliminate excessive ROS leads to progressive dysfunction of cellular organelles [157,158,159,160]. In the diabetic *db*/*db* mouse model, increased glial activation and apoptosis were observed in the ganglion cell layer [161].

## 4. Protective Role of Mitochondrial Uncoupling Proteins in T2D

UCPs are a family of mitochondrial anion carrier proteins located in the inner mitochondrial membrane that are encoded by SLC25 genes [162]. They are known to regulate glucose and lipid metabolism [35]. UCPs transport protons (H^+^) to the mitochondrial matrix, thereby dissipating the proton motive force and uncoupling ATP synthesis from substrate oxidation [34].

**Figure 2:**
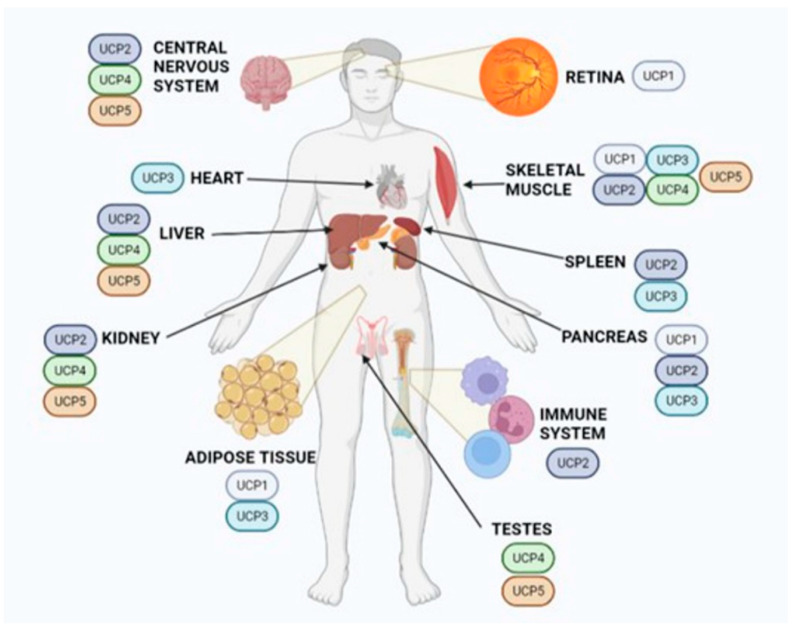
Uncoupling proteins (UCPs) are located in the mitochondria of many tissues. Apart from having tissue-specific roles, UCPs are known for their protective antioxidative activity. See the main text for details. Created with BioRender.com.

Five different UCPs have been identified thus far in different tissues (Figure 2). UCP4 and UCP5 are primarily expressed in the central nervous system and play an important role in brain metabolism and the development of central nervous system diseases [163,164,165]. During early life, UCP expression is similar in mice of both sexes. However, after puberty and throughout adulthood, there is a sexual dimorphism in the expression of UCP1 and UCP3 that correlates with weight gain. In males, the expression of UCP1 and UCP3 decreases with age. In females, however, the pattern of their expression is more variable, decreasing in young adulthood and increasing later [166]. This differential UCP1 and UCP3 expression pattern in male and female mice may explain the age-related weight gain between the sexes. During aging, males gain weight faster and to a greater extent than females. Overexpression of UCP1 and UCP3 in brown adipose tissue and skeletal muscle appears to mimic endurance training and prevent the development of obesity in female mice by reducing triglyceride accumulation [166,167,168].

Mitochondrial superoxide production strongly depends on the electrochemical proton gradient. Decreasing the electrochemical proton gradient and local oxygen availability limit the formation of ROS [169]. Accordingly, in oxidative stress conditions such as T2D, ischemia-reperfusion injury, and aging, uncoupling of mitochondrial metabolism can have a cytoprotective effect [170].

Mitochondrial UCPs can control mitochondrial ROS production by reducing the efficiency of oxidative phosphorylation [171,172]. Thus, inducible proton leakage through UCPs is an essential mechanism for controlling mitochondrial ROS generation by adjusting the electrochemical proton gradient [46,173,174]. In diabetic and obese individuals, alterations in glucose metabolism and the development of some pathologies of insulin signaling have been associated with specific gene polymorphisms of UCPs [37]. Accordingly, the hyperglycemia-induced increase in ROS production in aortic endothelial cells was completely abolished when the inhibitor of the respiratory chain complex II thenoyltrifluoroacetone (TTFA) or the uncoupler of oxidative phosphorylation carbonyl cyanide m-chlorophenylhydrazone (CCCP), which abolishes the proton gradient, was applied. The same effect was obtained by overexpressing UCP1 or manganese superoxide dismutase (MnSOD), suggesting that the damage caused by hyperglycemia-associated biochemical pathways (PKC, AGEs, and the polyol pathway) can be prevented by normalizing mitochondrial ROS levels [86].

In this review, we specifically focus on the protective role of UCPs in T2D, particularly UCP1, UCP2, and UCP3.

### 4.1. UCP1

Uncoupling protein 1 (UCP1) is mainly found in the mitochondria of adipose tissue but also in muscle, retinal cells, and the pancreas [175,176,177,178]. In general, the activity of UCP1 lowers membrane potential, reduces the generation of ROS, and increases energy expenditure and nonshivering thermogenesis [179], making it a candidate gene involved in the pathogenesis of T2D. The role of UCP1 is tissue-specific [180], as its ability to increase glutathione levels and reduce the production of ROS is far greater in skeletal muscle [179] than in brown adipose tissue, where its primary role is heat production [181]. Experiments in transgenic mice with skeletal muscle-specific UCP1 expression have shown a reduced incidence of age-related diseases and prolonged survival compared to wild-type mice [182]. Proteins involved in the coordination of metabolism, stress responses, and disease susceptibility were differentially affected in skeletal muscle-specific UCP1-expressing mice. Muscle levels of phosphorylated AMPK, a sensor of energy status in cells that helps maintain energy stores by regulating anabolic and catabolic pathways [183], were higher than in wild-type mice. On the other hand, the levels of phosphorylated mTOR, a serine/threonine kinase that plays an important role in anabolic and catabolic signaling, protein synthesis, and skeletal muscle remodeling [184], were lower than those in wild-type mice. Both changes in protein activity mimic caloric restriction, which has been associated with slower aging and age-related diseases [185]. In addition, uncoupled mice displayed a decrease in adipose tissue mass and serum IGF-1 and an increase in serum adiponectin [182], which was accompanied by a decrease in inflammation. Age-related diseases such as T2D are characterized by chronic inflammation, and adipose tissue is known to recruit inflammatory cells during obesity [186]. Increased adiposity is also associated with sympathetic nervous system activation and increased renal sodium reabsorption, leading to hypertension [187]. In transgenic mice with increased skeletal muscle-specific UCP1 expression, UCP1 activity showed great potential to reduce obesity and inflammation by accelerating skeletal muscle metabolism and energy expenditure. By reducing sympathetic nervous system activation and decreasing the secretion of norepinephrine, UCP1 induction in skeletal muscle increased urinary sodium excretion and lowered blood pressure [182].

### 4.2. UCP2

Mitochondrial uncoupling protein 2 (UCP2) is found in the muscle, spleen, pancreas, kidneys, central nervous system, and immune system. Its expression is stimulated by ROS and increases significantly with oxidative stress, playing an important antioxidant role [188,189,190]. The activity of UCP2, like that of UCP3, is controlled by glutathionylation and ROS-induced deglutathionylation [191]. This process involves the formation of mixed disulfides between glutathione and cysteine thiols of UCPs in response to oxidative or nitrosative stress, which regulates mitochondrial metabolism [191,192,193].

One of the first indications that UCPs play a role in reducing mitochondrial ROS production emerged from work by Negre-Salvayre et al. [172]. The authors determined that inhibition of UCP2 resulted in an increased electrochemical proton gradient and peroxide levels in mitochondria from the liver, spleen, and thymus. Subsequent studies using UCP2 knockout mice of different background strains [194] confirmed the observations of Negre-Salvayre, as the mice exhibited chronic oxidative stress. UCP2 overexpression studies confirmed its essential role in reducing oxidative stress, as the production of ROS was successfully reduced [195,196].

Several lines of evidence support a role for UCP2 in T2D. For instance, polymorphisms in the promoter regions of the *UCP2* gene have been associated with an enhanced risk for T2D in obese subjects [197] and variations in insulin secretion in glucose-tolerant subjects [198]. UCP2 is also abundantly expressed in pancreatic beta cells, particularly those of diabetic mice [199,200]. Increased UCP2 activity leads to proton leakage in the mitochondrial membrane, resulting in decreased ATP synthesis and ROS formation [172] and, at the same time, downregulation of glucose-stimulated insulin secretion [201,202,203]. In contrast, UCP2-deficient mice displayed increased insulin secretion [199].

Diabetes-associated hyperglycemia seems to play a critical role in regulating the expression of UCP2. In vitro studies of cells grown under chronically elevated glucose conditions (30 mmol/L) have shown that glucose-induced oxidative stress upregulates UCP2 [204]. Similarly, increased UCP2 activity has been observed in isolated mitochondria under non-phosphorylating and phosphorylating conditions of high glucose (25 mmol/L) [205]. This increased UCP2 activity affected the mitochondrial respiratory rate, mitochondrial membrane potential, and ROS generation. UCP2 activity attenuated ROS production by lessening the reduction level of mitochondrial respiratory chain complexes, resulting in increased antioxidant efficiency [205]. The importance of UCP2 uncoupling in endothelial stress resistance was established by He et al. (2014). Lentivirus-induced UCP2 overexpression was able to successfully inhibit apoptosis elicited by high glucose levels [206]. Thus, UCP2 acts as an essential sensor and negative regulator of mitochondrial ROS overproduction in response to hyperglycemia [205].

Moreover, high glucose exposure has been found to shift aerobic cell metabolism from carbohydrate oxidation to lipid and amino acid oxidation [207]. The resulting increase in mitochondrial oxidation of fatty acids decreases free fatty acid (FFA) concentrations, which protects the mitochondrial respiratory chain from the inhibitory effects of excessive amounts of fatty acids. The upregulation of UCP2 caused by the same hyperglycemic conditions makes the mitochondrial respiratory chain less sensitive to inhibition by FFAs [208].

Given its role in regulating insulin secretion and beta cell dysfunction, UCP2 may be a promising therapeutic target for the treatment of T2D. Accordingly, animal models of T2D treated with an antisense oligonucleotide against UCP2 showed improved insulin secretion and peripheral insulin action [209]. Similarly, Zhang et al. described a small molecule, genipin, that inhibited proton leakage mediated by UCP2 and stimulated insulin secretion from pancreatic islet cells [210].

Oxidative stress plays a key role in the development of diabetic retinopathy. Under hyperglycemic conditions, retinal cells respond to increased production of ROS with increased expression of the *UCP2* gene [211]. Significantly increased UCP2 expression was reported in the retinal cells of young adults of the diabetic *db*/*db* mouse model [212]. However, UCP2 expression decreases with age, making the *UCP2* gene a potential target for retinal cell protection against age-related ROS [213].

While the adverse effects of elevated glucose concentrations on retinal pathophysiology are well known, antioxidant therapy for diabetic retinopathy has shown limited benefit [153]. Since UCP2 plays a role in reducing the production of ROS, selective activation of UCP2 may have therapeutic potential in patients with diabetic retinopathy [214].

### 4.3. UCP3

Uncoupling protein 3 (UCP3) is expressed in the pancreas, skeletal muscle, heart, adipose tissue, and spleen. Its abundance in brown adipose tissue correlates with the abundance of UCP1 and is much greater than in skeletal muscle [215,216]. Indeed, UCP3 is the predominant UCP homolog in skeletal muscle [173], where it is critically involved in the metabolism of FFAs [217,218]. Lipids induce oxidative stress in mitochondria when their supply exceeds the oxidative capacity of mitochondria. Under such circumstances, UCP3 levels are upregulated by deglutathionylation [191]. Increased UCP3 levels have a protective effect by increasing the oxidative capacity of mitochondria, reducing the concentration of FFAs in the matrix [219], and decreasing the formation of ROS, which together have a positive effect on muscle insulin sensitivity [220]. Once the oxidative capacity of mitochondria is improved, the levels of UCP3 are downregulated [221].

Similar to UCP2, the degradation of UCP3 is rapid [222], allowing efficient adjustment of its level to rapidly changing metabolic needs and varying rates of ROS production during mitochondrial oxidative processes. The half-life of the UCP3 protein is approximately 60 times shorter than the half-life of the UCP1 protein [222].

**Figure 3:**
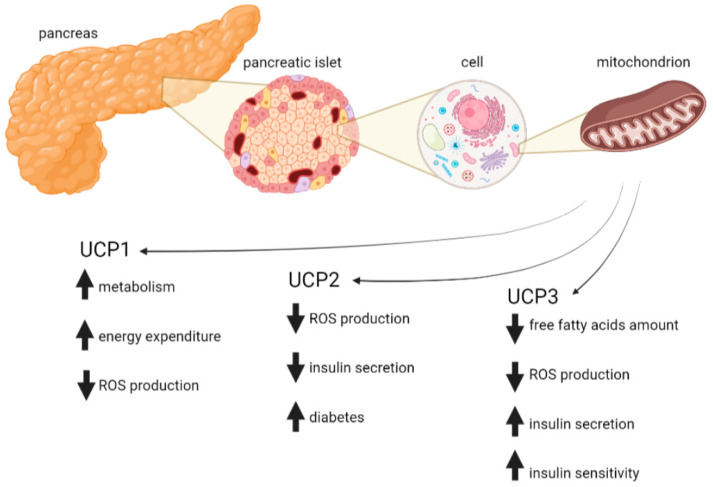
Role of pancreatic UCPs in T2D. The primary role of UCP1 in brown adipose tissue is heat production in a process known as nonshivering thermogenesis. Increased metabolism in pancreatic cells expressing UCP1 increases energy dissipation and reduces mitochondrial ROS production, thereby reducing oxidative stress and the progression of T2D. UCP2 acts as an antioxidant whose expression is stimulated by ROS. Increased UCP2 activity leads to increased proton leakage, which in turn decreases ATP synthesis and the formation of ROS. At the same time, it reduces glucose-stimulated insulin secretion, which may exacerbate T2D. UCP3 affects insulin secretion differently than UCP2. When UCP3 is activated, it reduces ROS production and increases fatty acid oxidation and insulin secretion. Created with BioRender.com.

Pancreatic beta cells express UCP3, which affects insulin secretion differently than UCP2 (Figure 3). When UCP3 is activated, it reduces ROS production and increases fatty acid oxidation and insulin secretion [223]. The role of the *UCP3* gene in the development of T2D and obesity has been intensively studied, and several *UCP3* gene polymorphisms have been reported to be associated with T2D [224,225,226]. Decreased expression of *UCP3* in skeletal muscle and pancreas has been found in diabetic patients [227].

## 5. Conclusions

UCPs are important regulators of energy homeostasis. They are closely related to aging and T2D, which are caused in part by the accumulation of oxidative damage. Moreover, some UCP polymorphisms correlate with human longevity and increased susceptibility to metabolic changes leading to T2D. By uncoupling mitochondrial energy production, UCP1 increases energy expenditure in adipose tissue, thereby decreasing adiposity and inflammation. Its role in increasing antioxidant glutathione levels in skeletal muscle appears to be even more important, making it a good candidate for developing interventions to treat T2D.

Under conditions of high oxidative stress and thus high ROS concentrations, the expression and activation of UCP2 increase, reducing ATP generation and insulin secretion in pancreatic beta cells. Since ATP is essential for closing ATP-dependent potassium channels and increasing insulin secretion from beta cells [108], pancreas-specific downregulation of UCP2 could be a potential pharmacological approach to keep insulin secretion sufficiently high to cope with elevated blood glucose concentrations in diabetic patients. At the same time, upregulation of UCP2 appears to be beneficial in tissues other than the pancreas by reducing the production of ROS via increased proton leakage in the mitochondrial membrane. Therefore, selective upregulation of UCP2 in these tissues could also be a potentially suitable therapeutic approach.

Finally, UCP3 reduces mitochondrial FFA concentration and thus decreases ROS generation. In diabetic patients, UCP3 expression is reduced, which negatively affects insulin sensitivity. Conversely, UCP3 activation increases insulin secretion. However, UCP3 is rapidly degraded in the liver, which may pose problems in using its upregulation as a potential strategy for T2D management.

Due to their antioxidant functions and ability to modulate insulin secretion and sensitivity, UCPs have great potential as targets for the development of new therapies against T2D and associated complications.
